# Factors promoting collaboration between community sports leaders and guardians in urban areas of Japan: A cross-sectional study

**DOI:** 10.3389/fpubh.2022.940580

**Published:** 2022-08-29

**Authors:** Yoshino Hosokawa, Hiroko Yako-Suketomo, Kaori Ishii, Koichiro Oka

**Affiliations:** ^1^Graduate School of Sport Sciences, Waseda University, Saitama, Japan; ^2^Faculty of Physical Education, Japan Women's College of Physical Education, Tokyo, Japan; ^3^Faculty of Sport Sciences, Waseda University, Saitama, Japan

**Keywords:** community sports leader (CSL), coordination function, regional sports, guardian, Japan

## Abstract

**Background:**

Community sports leaders (CSLs) are expected to play an important role in promoting regional sports in Japan. Increased opportunities for CSLs to work in schools and engage with guardians could encourage middle-aged adults to participate in regional sports activities. However, few CSLs work with guardians, and there is no evidence regarding what CSL characteristics encourage collaboration. The present study aimed to ascertain the aspects of coordination for CSLs collaborating with guardians in urban areas.

**Methods:**

A self-administered questionnaire survey was mailed to all 1,489 CSLs through 59 municipalities using the CSL out of 62 in Tokyo, Japan, from July to November 2021. The questionnaire covered sociodemographic data, variables related to CSL activities, coordination functions, and experience collaborating with guardians. We conducted a binomial logistic regression analysis using experience collaborating with guardians as the dependent variable, coordination function as the explanatory variable, and gender, age, residence duration, years of CSL experience, and the number of activities as covariates.

**Results:**

The analyzed sample comprised 478 CSLs. Binomial logistic regression analysis revealed a significant relationship between experience collaborating with guardians and the coordination function of internal/external CSL organizations, such as participation in non-CSL community activities, experience teaching and supporting their own children's sports, activity initiatives, number of times they used the school gymnasium, awareness of the regional sports plan, and cooperation with the chairman of the neighborhood association.

**Conclusions:**

Collaboration with guardians was related to CSL's individual experience engaging in regional sports from parenting and community collaboration, such as participation in non-CSL community activities and their relationship with the chairman of the neighborhood association. CSL activities may have the potential to encourage middle-aged adults to participate in regional sports.

## Introduction

Although exercise and sports effectively prevent the onset of metabolic syndrome and related diseases, which are common among middle-aged adults (1–4), the rate of engaging in sports among middle-aged adults remains lower than that of other generations (5). A national survey in Japan reported that the main reason for the interruption of exercise or sports was being occupied with work and daily life (6). Regular employees in Japan have long working hours (7), and middle-aged adults who prioritize raising children may restrict their opportunities to participate in exercise or sports (8–10). For this reason, the Sports Basic Plan of the Ministry of Education, Culture, Sports, Science and Technology emphasizes enhancing sports opportunities for working and child-rearing generations as an essential issue (11).

The decision to host the Tokyo Olympic and Paralympic Games enhanced sports education as well as the development of sports facilities in Japan. In addition, the development of tangible human resources to support regional sports activities played an important role. A typical example is sports-focused volunteers called community sports leaders (CSLs), who promote regional sports (12). CSLs are community members who engage in activities creating a bridge between community members and local governments (11, 12). Community-based volunteers with similar roles have been referred to as community health workers, village health workers, community health advisors, and lay health advisors in health promotion literature in other countries (13). The practical use of such community-based volunteers effectively improves community health through social interaction (14). Community-based volunteers have played a significant role in public health in Japan. Originally, there were various other volunteers, such as health volunteers (15–19), district welfare commissioners (20), and voluntary disaster-prevention organizations (21), engaged in community-based activities according to their purposes. Several previous studies in Western countries have shown that community-based volunteer support for exercise and sports can cause neighborhood residents to increase activity levels (22–24). These findings support the usefulness of the Japanese CSL.

In Japan, CSL organizations were established in 1957 under the direction of the national government for providing physical education programs to the residents. Most of them were active workers, and there were more men than women in the CSL Association. Some people become CSLs on their own initiative, whereas others are invited to perform this role. Training classes are regularly held in each municipality, and the municipal budgets include all CSL projects. In addition, CSLs are rewarded for their labor by the local government (25). The main activities of CSLs include organizing sports events at public community facilities and teaching sports to children at the request of schools (25). In addition, the Sports Basic Law enacted in 2011 (12) established a new role for the CSL as a coordinator of regional sports projects. Previous studies show that CSLs have coordination functions consisting of internal and external organizational factors: social resource utilization, project development, and organic collaboration with stakeholders involved in regional sports activities (26). The collaborative relationship between CSLs and guardians is especially essential when organizing sports projects for neighborhood children and using the school gymnasium (27, 28). In other words, increased collaboration between CSLs and guardians could improve opportunities for middle-aged adults to participate in regional sports.

However, interpersonal problems sometimes occur between children's sports coaches and guardians, such as distrust due to a lack of communication (29, 30). Previous studies have reported that some CSLs maintain good interpersonal relationships with diverse organizations, including guardians, and collaborate to provide regional sports programs (26). Considering the relationship between CSLs and guardians requires understanding the characteristics of CSLs who collaborate with their guardians.

Therefore, the present study aimed to ascertain the aspects of coordination among CSLs collaborating with guardians in urban areas. By clarifying the characteristics of functions that promote collaboration between CSLs and guardians, local governments considering measures to promote regional sports for middle-aged adults could find effective ways to use CSLs.

## Methods

### Setting and research participants

This study had a cross-sectional design. The participants were all 1,489 CSLs (892 men and 597 women) registered in 2021 with the CSL association in Tokyo, Japan. An anonymous self-administered questionnaire survey was conducted by mail in 59 municipalities using the CSL out of 62 municipalities in Tokyo from July to November 2021. Questionnaires were distributed to all active CSLs through each local government sports department administrator and returned by mail directly to the author's affiliated institution from each CSL.

### Coordination function measures

The items related to coordination functions were determined by referring to previous CSL studies and earlier studies on health promotion volunteers in Japan (15–19, 26). We organized these variables into internal and external organizational factors based on previous studies that conducted interviews with CSLs regarding their coordination function (26). To verify the reliability of the variables for the CSLs' coordination function, intraclass correlation coefficients and weighted Cohen's kappa coefficients were calculated using the re-test method at 1-month intervals from one municipality to check the degree of agreement in the responses. Twenty of the 24 respondents completed the 1-month re-test survey (83.3%). For democratic organizational activities (four items) included in the internal organizational factors, the intraclass correlation coefficient was 0.823 (95% confidence intervals [CI]: 0.648–0.921). The other 15 variables related to internal/external organizational factors had weighted Cohen's kappa coefficients were 0.565–1.000. Generally, reliability is proven when the intraclass correlation coefficient is 0.75 to 0.9 or higher (31) and Cohen's kappa coefficient is around 0.4 to 0.6 or higher (32).

#### Internal organizational factors

Participants were asked about participation in non-CSL community activities (had participated/had not participated) and whether they had taught and supported their own children's sports (had experienced or had not experienced). Activity initiatives were classified into two categories: helping other organizations and planning and managing independently. They reported the number of times they used the school gymnasium and were categorized into two types: < once a month (never [= 1], < once every 2 months [= 2], ≥ once every 2 months to < once a month [= 3]), and ≥ once a month (≥ once a month to < two times a month [= 4], ≥ two times a month to < four times a month [= 5], ≥ four times a month [= 6]). Regarding awareness of the regional sports plan, we dichotomized “I know the name and meaning” and “I know the name but not the meaning” or “I don't know the name.” They answered the burden bias among CSLs using a six-point Likert scale and were categorized into two groups: I disagree (strongly disagree [= 1], disagree [= 2], disagree a little [= 3]) and agree (agree a little [= 4], agree [= 5], strongly agree [= 6]). They assessed communication with community organizations and democratic organizational activities on a five-point Likert scale (strongly disagree [= 1], disagree a little [= 2], neither agree nor disagree [= 3], agree a little [= 4], strongly agree [= 5]). Communication with community organizations was categorized into two groups (1–3 and 4–5). Scores for democratic organizational activities were summed (score range: 4–20). The higher the score, the more democratic the organizational activities (19). In the present study, Cronbach's alpha for the total score was 0.862. The total score of democratic organizational activities was dichotomized at the median for analysis (high score ≥ 16/low score < 16).

#### External organizational factors

Participants were asked about their experience of collaborating with guardians (collaboration with guardians/no collaboration with guardians). They reported their collaboration with local government (sports department, other departments) and with community organizations (sports-related, non-sports-related) on a five-point Likert scale (strongly disagree [= 1], disagree a little [= 2], neither agree nor disagree [= 3], agree a little [= 4], strongly agree [= 5]). Collaboration with local governments and community organizations was classified into two categories (1–3 or 4–5). Regarding neighbors' perceptions of CSL and cooperation with neighborhood schools, respondents answered on a four-point Likert scale and dichotomized them into two groups: agree (agree [= 1], agree a little [= 2]) and disagree (disagree a little [= 3], disagree [= 4]). They assessed their cooperation with the chairman of the neighborhood association and were categorized into two groups: I agree (agree [= 1], agree a little [= 2]) and disagree (disagree a little [= 3], disagree [= 4], and no neighborhood association [= 5]).

### Sociodemographic and CSL activity-related measures

We asked about gender (men/women), age (in years), employment status (employed/unemployed), educational level (high school graduate or less/junior college graduate or equivalent/college graduate or higher), living arrangement (living alone or with others), household income (< 5,000,000 yen/ < 10,000,000 yen/≥ 10,000,000 yen), administrative unit (city/ward/town/village), and residence duration (years).

CSL activity-related measures included reasons for becoming a CSL (active/passive), number of activities participated in over 6 months (< once a week/≥ once a week), annual compensation (< 100,000 yen/≥ 100,000 yen), board experience (current or past experienced/no experience), and years of CSL experience (years).

### Statistical analysis

First, chi-square tests and *t*-tests were performed to examine the proportional and average differences between sociodemographic and CSL activity-related measures, and the experience of collaborating with guardians.

Moreover, a binomial logistic regression analysis examined the relationships between experience collaborating with guardians and all explanatory variables. In the first model (Model 1), univariate logistic regression analysis was used to provide unadjusted odds ratios (OR) and 95%CI to determine the relationship between the experience of collaborating with guardians and each of the explanatory variables. We used gender, age, residence duration, years of CSL experience, number of activities participated in over 6 months, and board experience as covariates in model 2. In model 2, the adjusted odds ratios (AORs) and 95%CIs were calculated. We used a variance inflation factor (VIF) to confirm that multicollinearity did not occur among the explanatory variables (VIF < 10). The model's goodness of fit was also checked using the Hosmer-Lemeshow test. The level of significance for all analyses was set at *p* < 0.05. Statistical analyses were performed using SPSS version 26.0 J for Windows (Statistical Package for the Social Sciences; SPSS Inc. Chicago, IL, USA).

## Results

Questionnaires were distributed to 1,276 CSLs among 1,489 total CSLs. Nine municipalities did not cooperate with the survey (*n* = 136), and several CSLs were unable to receive their questionnaires during the survey period (*n* = 77). A total of 571 questionnaires were returned (response rate, 44.7%). Eighty-eight CSLs who became CSLs after 2020 were excluded from the analysis because they had little or no activity due to COVID-19. Additionally, we excluded five CSLs who did not have answers regarding experience collaborating with guardians, and 478 were used for the analysis (valid response rate, 37.5%).

Table 1 presents the sociodemographic characteristics of the study participants. The mean age (SD) was 58.9 (10.4) years; 59.4% were men, 79.3% were employed, 50.8% had graduated from 4 years of university or higher, and 9.0% were living alone. Overall, 37.0% of the respondents had a household income of < 5,000,000 yen per year, whereas 15.3% earned more than 10,000,000 yen per year. Moreover, 60.3% of the participants reported collaborating with guardians. Significant proportional differences in experience collaborating with guardians were observed for several items. The group that collaborated with guardians had a higher percentage of those who were older (*p* = 0.012) and had lived in the area longer (*p* = 0.015). In the CSL activity-related measures, the group collaborating with guardians had a higher percentage of those who had been active as CSLs for a long time (*p* = 0.004), participated in activities at least once a week (*p* = 0.005), and had experience as a board member (*p* = 0.001).

**Table 1:** Characteristics of CSLs with and without the collaboration of guardians.

	**Total (*****N*** = **478)**		**Collaboration with guardians** **(*****n*** = **288)**		**No collaboration with guardians** **(*****n*** = **190)**		***p***
	***n***	**(%)**	***n***	**(%)**	***n***	**(%)**	
Gender							
Men	284	(59.4)	174	(60.4)	110	(57.9)	0.492
Women	191	(40.0)	111	(38.5)	80	(42.1)	
Employment status							
Employed	379	(79.3)	234	(81.3)	145	(76.3)	0.289
Unemployed	95	(19.9)	53	(18.4)	42	(22.1)	
Educational level							
College graduate or higher	243	(50.8)	149	(51.7)	94	(49.5)	0.467
Junior college graduate or equivalent	113	(23.6)	62	(21.5)	51	(26.8)	
Less than high school graduate	117	(24.5)	72	(25.0)	45	(23.7)	
Living arrangement							
Living alone	43	(9.0)	22	(7.6)	21	(11.1)	0.226
Living with others	427	(89.3)	259	(89.9)	168	(88.4)	
Household income							
< ¥5,000,000	177	(37.0)	102	(35.4)	75	(39.5)	0.605
< ¥10,000,000	201	(42.1)	126	(43.8)	75	(39.5)	
≥ \10,000,000	73	(15.3)	44	(15.3)	29	(15.3)	
Administrative unit							
City	197	(41.2)	110	(38.2)	85	(44.7)	0.125
Ward	260	(54.4)	167	(58.0)	93	(48.9)	
Town	15	(3.1)	6	(2.1)	9	(4.7)	
Village	6	(1.3)	3	(1.0)	3	(1.6)	
Reasons for becoming a CSL							
Active	51	(10.7)	32	(11.1)	19	(10.0)	0.759
Passive	423	(88.5)	256	(88.9)	167	(87.9)	
The number of activities participated in over 6 months				
< Once a week	353	(73.8)	201	(69.8)	152	(80.0)	0.005**
≥ Once a week	113	(23.6)	81	(28.1)	32	(16.8)	
Annual compensation							
< ¥100,000	257	(53.8)	159	(55.2)	98	(51.6)	0.630
≥ ¥100,000	158	(33.1)	94	(32.6)	64	(33.7)	
Board experience							
No experience	206	(43.1)	101	(35.1)	105	(55.3)	<0.001**
Current or past experienced	271	(56.7)	186	(64.6)	85	(44.7)	
	**Mean**	**(** ***SD*** **)**	**Mean**	**(** ***SD*** **)**	**Mean**	**(** ***SD*** **)**	
Age	58.9	(10.4)	60.0	(8.9)	57.4	(12.2)	0.012*
Residence duration	36.6	(18.7)	38.3	(18.4)	34.1	(18.8)	0.015*
Years of CSL experience	11.4	(7.7)	12.3	(7.5)	10.2	(7.8)	0.004**

CSL, community sports leader,

^*^p < 0.05,

^**^p < 0.01,

chi-square test and t-test.

Table 2 shows the results of the binomial logistic regression analysis that examined the factors associated with the experience of collaborating with guardians. Regarding internal organizational factors, having participated in non-CSL community activities (AOR: 2.76; 95%CI: 1.09–6.97) and experienced teaching and supporting their own children's sports (AOR: 2.92; 95%CI: 1.93–4.44) were positively associated with collaborating with guardians for CSL. Planning and managing independently (AOR: 0.57; 95%CI: 0.34–0.95) were negatively associated with collaborating with guardians in CSL. In addition, using the school gymnasium at least once a month (AOR: 1.74; 95%CI: 1.16–2.59) and awareness of the regional sports plan (AOR: 2.17; 95%CI: 1.44–3.28) were positively associated with collaboration with guardians for CSL. Finally, cooperation with the chairman of the neighborhood association (AOR: 1.74; 95%CI: 1.17–2.58) was positively associated with collaborating with guardians in external organizational factors. The Hosmer-Lemeshow test showed *p* ≥ 0.05 for all measures of coordination function, indicating a good fit for the model.

**Table 2:** Factors associated with collaboration with guardians: Binomial logistic regression analysis.

	**Model 1**		**Model 2**	
	**OR**	**(95%CI)**	**AOR**	**(95%CI)**
Internal organizational factors
Participation in non-CSL community activities				
I had not participated in non-CSL community activities	1	(ref)	1	(ref)
I had participated in non-CSL community activities	3.65	(1.55–8.58) **	2.76	(1.09–6.97) *
Whether they had taught and supported their own children's sports
I had not experienced	1	(ref)	1	(ref)
I had experienced	2.95	(1.99–4.38) **	2.92	(1.93–4.44) **
Activity initiatives
Helping other organizations	1	(ref)	1	(ref)
Planning and managing independently	0.65	(0.40–1.04)	0.57	(0.34–0.95) *
Number of times they used the school gymnasium
< Once a month	1	(ref)	1	(ref)
≥ Once a month	1.98	(1.36–2.89) **	1.74	(1.16–2.59) **
Awareness of the regional sports plan
I don't know	1	(ref)	1	(ref)
I know the name and meaning	2.62	(1.79–3.82) **	2.17	(1.44–3.28) **
Burden bias among CSLs
I disagree	1	(ref)	1	(ref)
I agree	0.79	(0.55–1.15)	0.80	(0.54–1.19)
Communication with community organizations
I disagree	1	(ref)	1	(ref)
I agree	0.97	(0.67–1.40)	1.00	(0.68–1.49)
Democratic organizational activities (range: 4–20)
<16 (Low)	1	(ref)	1	(ref)
≥ 16 (High)	0.96	(0.66–1.40)	0.96	(0.64–1.43)
External organizational factors
Collaboration with local governments (sports department)
I disagree	1	(ref)	1	(ref)
I agree	1.13	(0.65–1.97)	1.07	(0.60–1.93)
Collaboration with local governments (other department)
I disagree	1	(ref)	1	(ref)
I agree	1.54	(1.07–2.24) *	1.39	(0.94–2.06)
Collaboration with community organizations (sports-related)
I disagree	1	(ref)	1	(ref)
I agree	0.88	(0.60–1.30)	0.92	(0.61–1.40)
Collaboration with community organizations (non-sports-related)
I disagree	1	(ref)	1	(ref)
I agree	1.05	(0.73–1.52)	1.00	(0.67–1.48)
Neighbors' perceptions of CSL
I disagree	1	(ref)	1	(ref)
I agree	1.12	(0.74–1.69)	1.07	(0.69–1.66)
Cooperation with neighborhood schools
I disagree	1	(ref)	1	(ref)
I agree	1.20	(0.82–1.75)	1.20	(0.80–1.79)
Cooperation with the chairman of the neighborhood association
I disagree	1	(ref)	1	(ref)
I agree	1.67	(1.16–2.43)**	1.74	(1.17–2.58)**

CSL, community sports leader; 95% CI, 95% confidence interval; OR, odds ratio; AOR, adjusted odds ratio. Model 2: Adjusted for gender, age, residence duration, years of CSL experience, number of activities participated in over 6 months, and board experience.

^*^p < 0.05,

^**^p < 0.01.

## Discussion

This study suggests that collaboration between CSLs and guardians is related to the potential characteristics of individual CSLs and their community relationships. The CSLs collaborating with guardians tended to have been active as CSLs for a long time and made a greater contribution to CSL activities. Experienced veterans are more likely to establish reciprocal cooperative relationships with community members (33). These results support previous research showing that years of volunteering are associated with increased community outreach (17). Independent of these characteristics, one of the essential findings in this study is that CSLs who had experience with teaching and supporting their own children's sporting activities before becoming CSLs had 2.92 times greater odds of collaborating with guardians of neighborhood children after their children had left school. Previous studies have found that children's participation in sports influences their guardians' network, and guardians' interest in sports support our findings in part (27, 28). Another study reported that the experience of positive interaction among guardians in middle age was associated with higher levels of community consciousness in old age (34). While diverse interactions among guardians during the parenting period are valuable in forming guardians' community attitudes, few guardians are involved in their children's sports (34). This result suggests that the experience of actively interacting with other guardians by teaching and supporting their own children's sports may have contributed to their becoming CSLs.

We also found that CSLs who used the school gymnasium at least once a month had 1.7 times greater odds of collaborating with guardians than those who reported using it less frequently. Lack of time and an environment for sports are often barriers preventing middle-aged adults from participating in sports and a disincentive for sports leaders to become involved in schools (8–10, 29). However, it has been reported that sports leaders being guardians of children and trusted by stakeholders facilitate the involvement of sports leaders in children's sports activities (35). CSLs who continuously engaged in regional sports through their children's activities might have had an easier time using the school gymnasium (29, 35). Our findings show the importance of intersectoral collaboration in community organizations, such as participation in other community activities and the relationship with neighborhood association chairman. A previous study of Japanese health volunteers stated that cooperation with neighborhood associations makes it easier for residents to reach out (18). Similarly, CSLs might have contacted guardian groups through community organizations, which residents trust. Thus, CSLs promote regional sports programs by regularly engaging with social resources.

The CSLs who collaborated with guardians in this study tended to believe they were helping other organizations more than taking the initiative to plan and manage their activities. In addition, CSLs with experience collaborating with guardians were more likely to be aware of regional sports plans. According to Brunton et al.'s community empowerment framework (36), community members involved in diverse health promotion activities, possessing high communication and leadership skills, will become involved in decision-making and planning design of local governments. Farmer et al. (37) argued that partnerships between residents, who understand local issues, context, and feasible local solutions, and government policymakers, who provide community services, can effectively diffuse grassroots activities. This study's results can also be explained as expressing aspects of CSLs as partners with government policymakers. Consequently, grassroots outreach to guardians by CSLs is expected to encourage middle-aged adults to participate in regional sports.

Based on the literature on community empowerment frameworks (36), we predicted that CSLs engaged in democratic organizational activities would be more likely to proactively reach out to their communities and collaborate with guardians; however, we did not find any significant association among these factors. One explanation for this unexpected finding is that whether CSLs are available to collaborate with guardians depends mainly on their background, including their experiences in parenting their children. Moreover, the results suggest that a CSL organization's management system necessarily lead to organizational empowerment (36). Further research on the organizational management of CSLs is needed to ensure the sustainability of CSL activities so that the organization can maximize the capabilities of individuals.

## Strengths and limitations

This study is the first to suggest the characteristics of the coordination function among Japanese CSLs collaborating with guardians. This study has several strengths. First, it successfully conducted a questionnaire survey of all CSL associations in Tokyo. Our findings quantitatively demonstrate the characteristics of the overall coordination function of CSLs in urban areas, while a previous study on CSLs qualitatively investigated their community roles (26). Second, we showed that social interaction, which is the most crucial characteristic of CSLs, such as communication with community organizations outside of CSL, was an effective strategy for promoting sports to middle-aged adults who have few opportunities to participate in such activities. The findings here can be used for future CSL member recruitment and educational programs.

This study has several limitations. First, it was conducted only on Japanese CSLs, with the target CSL organizations limited to those in Tokyo. CSLs are original because their activities promote regional sports. Although this study provides practical evidence regarding ways to encourage CSLs, future studies should include different settings to increase the generalizability of the findings (e.g., provincial cities outside Tokyo). Second, study data were collected in 2020, the year of the COVID-19 pandemic. CSLs did not offer their usual activities during the survey period, so we inquired about activity before the COVID-19 pandemic. Furthermore, only 44.7% of CSLs returned a completed questionnaire. If only those with strong opinions on the topic responded, the results for this sample might have been biased. We must carefully interpret the findings, considering the data collection period, because measures of CSL activity may be subject to recall bias and selection bias. Nevertheless, this study confirms the efforts of CSLs to promote regional sports even during the COVID-19 pandemic.

## Conclusions

Collaboration with guardians was related to CSL's individual experience engaging in regional sports from parenting and community collaboration, such as participation in non-CSL community activities and their relationship with the chairman of the neighborhood association. CSL activities may have the potential to encourage middle-aged adults to participate in regional sports.

## Data availability statement

The datasets presented in this article are not readily available because all the relevant data are presented in this paper. The datasets used and/or analyzed during the current study are available from the corresponding author upon reasonable request. Requests to access the datasets should be directed to YH, yoshino.hosokawa@akane.waseda.jp.

## Ethics statement

The studies involving human participants were reviewed and approved by the Ethics Review Committee of Waseda University. The patients/participants provided their written informed consent to participate in this study.

## Author contributions

YH contributed to the design, implementation, data collection, data analysis, and writing of the manuscript. HY-S, KI, and KO provided input on the research idea and statistical analyses and edited the manuscript. All authors approved the final version of the manuscript and agreed to be accountable for all aspects of the work.

## Funding

KI was supported by a Grant-in-Aid for Scientific Research (No. 20K11473) from the Japan Society for Promotion of Science. KO was supported by a Grant-in-Aid for Scientific Research (No. 20H04113) from the Japan Society for Promotion of Science.

## Conflict of interest

The authors declare that the research was conducted in the absence of any commercial or financial relationships that could be construed as a potential conflict of interest.

## Publisher's note

All claims expressed in this article are solely those of the authors and do not necessarily represent those of their affiliated organizations, or those of the publisher, the editors and the reviewers. Any product that may be evaluated in this article, or claim that may be made by its manufacturer, is not guaranteed or endorsed by the publisher.
